# Elevated circulating homocysteine concentrations delayed nerve conduction velocity and increase the risk of diabetic kidney disease in patients with type 2 diabetes

**DOI:** 10.3389/fendo.2024.1451758

**Published:** 2024-12-11

**Authors:** Nannan Lv, Luzhu Jia, Fei Liu, Lan Cheng, Feng Liu, Jinsong Kuang, Xin Chen

**Affiliations:** ^1^ Department of Endocrinology and Metabolism, The Fourth People’s Hospital of Shenyang, China Medical University, Shenyang, China; ^2^ Department of Epidemiology, School of Public Health, Dalian Medical University, Dalian, China

**Keywords:** homocysteine, nerve conduction velocity, diabetic kidney disease, diabetic peripheral neuropathy, type 2 diabetes

## Abstract

**Introduction:**

China has the largest population of individuals with diabetes, and the prevalence of various complications among patients with type 2 diabetes remains high. Diabetic nephropathy affects approximately 20% to 40% of diabetic patients, becoming a major cause of chronic kidney disease and end-stage renal disease. Furthermore, around 50% of patients develop diabetic peripheral neuropathy (DPN), which is closely associated with physical disability, increased healthcare costs, and reduced work productivity. There is an urgent need for novel strategies in prevention, diagnosis, and treatment to improve patient outcomes.

**Methods:**

In this study, 163 patients with type 2 diabetes were selected as the observation group and further divided into three subgroups based on homocysteine (HCY) levels. The study measured several clinical parameters, including homocysteine, blood glucose, blood lipids, glycated hemoglobin, urinary microalbumin, urinary albumin-to-creatinine ratio (ACR), electromyography, and highly-sensitive C-reactive protein (CRP), among others. The levels of these indicators were analyzed and compared across the subgroups.

**Results:**

The results revealed significant differences in uric acid, creatinine, urinary microalbumin, urinary ACR, and nerve conduction velocity (right tibial nerve sensory conduction) among different HCY levels in patients with type 2 diabetes (P < 0.05). Linear regression analysis indicated that homocysteine levels were associated with systolic blood pressure, glycated hemoglobin, fasting C-peptide, uric acid, creatinine, urinary microalbumin, and nerve conduction velocity (including motor conduction velocity of the ulnar nerve and sensory conduction velocity of the sural nerve).

**Discussion:**

The clinical assessment of homocysteine in diabetic patients holds significant importance in the prevention of microvascular complications. Lowering HCY levels may offer a promising therapeutic approach for managing microvascular disease in diabetes.

## 1 Introduction

In recent years, the prevalence of diabetes has been steadily increasing both globally and in China. Epidemiological research conducted by Teng Weiping’s team shows that the prevalence of diabetes in China has risen from 9.7% in 2007 to 10.4% in 2017. According to the World Health Organization (WHO) criteria, 11.2% of Chinese adults aged 18 years and older had diabetes in 2017, with this figure rising to 12.8% when glycated hemoglobin (HbA1c) is included in the diagnostic criteria (1). Diabetes has thus emerged as one of the most serious global public health challenges. Survey results reveal that type 2 diabetes (T2DM) is the most prevalent form of diabetes in China, while the incidence of type 1 diabetes (T1DM) and other forms is comparatively lower. T1DM patients typically develop diabetic nephropathy within five years of disease onset, whereas T2DM patients may already have diabetic nephropathy at the time of diagnosis. Renal dysfunction in diabetic patients is significantly associated with an increased risk of all-cause mortality (2, 3). Diabetic peripheral neuropathy (DPN) is another common complication of T2DM, with its prevalence increasing as the duration of diabetes extends. DPN affects approximately half of all diabetes patients. Its clinical manifestations include numbness, pain, and abnormal sensations in the limbs, which in severe cases can lead to serious complications such as ulcers, infections, Charcot joints, foot or ankle fractures, amputations, and even depression. DPN-related pain can severely impact a patient’s sleep, emotional well-being, and overall physiological function, leading to a diminished quality of life (2, 4, 5). Given these challenges, early screening and intervention for diabetic nephropathy and peripheral neuropathy are crucial for improving patient outcomes and prognosis.

Homocysteine (HCY) is a sulfur-containing, non-protein amino acid in the human body and an important intermediate in the metabolism of methionine and cysteine. The main source of homocysteine in the human body is the absorption of methionine in animal protein (such as beef, mutton, pork, chicken, fish, eggs, etc.), so each human body has different levels and contents of homocysteine. The normal human body is tiny. In general, homocysteine levels are higher in men than women, and homocysteine levels increase with age. The normal level in the blood is generally 0-15 μmol/L when the protein level in the body is too high. or there is a lack of folic acid, vitamins B6 and B12, homocysteine levels can increase. When choosing health examinations, great importance is usually placed on blood sugar levels, blood lipids and uric acid in the blood, and homocysteine levels are often ignored. When homocysteine builds up in the blood, hyperhomocysteinemia (hHCY) occurs, and long-term hHCY damages the body’s cells, tissues, and organs. These are risk factors for the occurrence of many diseases. Elevated homocysteine is a known risk factor for systemic atherosclerosis and cardiovascular disease (CVD) and has been identified as a risk factor for several eye diseases such as diabetic retinopathy (DR) and age-related macular degeneration (AMD) (6). Recent advances have shown that elevated plasma Hcy is also a fundamental cause of neurodegenerative diseases (including Alzheimer’s disease, Parkinson’s disease and dementia), diabetes, Down syndrome and megaloblastic anemia. In recent years, it has also been shown that elevated plasma homocysteine levels are closely related to cancer (7).

The microvascular diseases of diabetes mainly include diabetic nephropathy, diabetic retinopathy, diabetic cardiomyopathy and diabetic neuropathy. Some studies have shown that homocysteine is independently associated with the prevalence of diabetic neuropathy in type 2 diabetes patients (8). The meta-analysis suggests that compared with type 2 diabetes patients without DPN, serum folate and vitamin B12 levels are lower in type 2 diabetes patients with DPN (9), and their reduction becomes an increase of homocysteine levels. At the same time, some studies have shown that hyperhomocysteinemia is an independent risk factor for the occurrence of diabetic peripheral neuropathy (10). There was also a study that suggested that HCY could be used for early prediction of dabetic nephropathy (11). However, no study has simultaneously observed an association between HCY and diabetic nephropathy and neuropathy. Currently, there are no effective means for early detection and treatment of microvascular complications in type 2 diabetes. There is an urgent need for new methods for prevention, diagnosis and treatment of DPN to change the prognosis of the disease. In this study, we verified the relationship between HCY and DN, DPN by observing homocysteine level, nerve conduction velocity, urinary ACR, blood creatinine and other indicators in patients with type 2 diabetes, to provide new ideas for early diagnosis of complications finding diabetes to scientifically guide the treatment of DN, DPN and reduce the economic burden. This study is a retrospective cross-sectional study. The main purpose of the study was to determine whether HCY is associated with microvascular complications such as DPN and DKD in type 2 diabetes. The secondary purpose was to determine whether HCY is associated with age, gender, blood pressure, BMI and other factors.

## 2 Patients and methods

### 2.1 Selection of patients and the research design

Inclusion criteria: ① Patients with type 2 diabetes mellitus (T2DM); ② Age over 18 years; ③ Body mass index (BMI) between 18.5 and 30 kg/m². Exclusion criteria: ① Patients with type 1 diabetes or other specific types of diabetes; ② Non-diabetic nephropathy patients with chronic kidney disease; ③ Patients with an estimated glomerular filtration rate (eGFR) less than 10 mL/min/1.73 m² requiring dialysis; ④ Patients with acute complications of diabetes, such as diabetic ketoacidosis or diabetic lactic acidosis; ⑤ Patients with recent (within 6 months) cerebrovascular accidents, such as cerebral infarction, or cardiovascular conditions, such as acute myocardial infarction and peripheral vascular obstructive disease; ⑥ Patients with acute infections, malignancies, or liver diseases; ⑦ Pregnant women. The diagnosis of T2DM was based on the 2014 American Diabetes Association guidelines, which define T2DM as a fasting plasma glucose level of ≥7.0 mmol/L, glycated hemoglobin (HbA1c) concentration of ≥6.5%, or a 2-hour postload plasma glucose concentration of ≥11.1 mmol/L (following a 75 g oral glucose tolerance test) (12). A total of 163 patients with T2DM from the Fourth People’s Hospital of Shenyang were included in the study. The patients were divided into three groups based on their homocysteine (HCY) levels: HCY < 10 µmol/L, HCY 10–15 µmol/L, and HCY > 15 µmol/L. This study was approved by the Medical Ethics Committee of the Fourth People’s Hospital of Shenyang (Approval No. 2021(11)-01), and all participants provided written informed consent.

### 2.2 Data collection

All patients were treated in the Department of Endocrinology and Metabolism of our hospital from July 2022 to December 2022. All study data was tested in hospitals and all tests were validated by reference laboratories. During the physical examination, body weight was measured with thin clothing and height without shoes. The body mass index (BMI) was then calculated using the formula weight/(height)^2^. Waist circumference was measured midway between the lower costal margin and the upper iliac crest in the central axis. Subjects’ basic information (gender, age, medical history, medication history, BMI, blood pressure, waist circumference, etc.) was recorded; Urine albumin/creatinine ratio (UACR), C-reactive protein (CRP), blood lipids including low-density lipoprotein (LDL), cholesterol (TCHOL), high-density lipoprotein (HDL), triglyceride (TG), serum creatinine (Scr), blood urea nitrogen (BUN), blood uric acid (UA), blood glucose, glycosylated hemoglobin (HbA1C), plasma HCY, electromyogram, ACR, carotid artery ultrasound were measured.

### 2.3 Definition and grouping of diabetic peripheral neuropathy and nephropathy

Distal symmetrical multiple neuropathy (DSPN) is the most representative neuropathy in diabetes. Their manifestations include bilateral pain in the distal symmetrical limbs, numbness, sensory abnormalities, etc. In this study, we divided patients into three groups based on their cumulative decrease in nerve conduction velocity (Descending Velocity <30%, 30–100%, >100%). Diabetic nephropathy (DN) is often based on a persistent high UACR increase and/or eGFR decrease, while other chronic kidney diseases (CKD) are excluded. Clinical diagnosis. In this study, three groups were divided according to urine ACR (ACR < 2.5 mg/mmol, ACR 2.5–30 mg/mmol, ACR > 30 mg/mmol).

### 2.4 Statistical analysis

Continuous variables were expressed as mean (SD) and categorical variables as number (percentage). Two group comparisons were performed using the t-test, Wilcoxon rank-sum test, or chi-square test when appropriate. To examine the relevance between homocysteine and other biomarkers, simple linear regression analysis and multiple linear regression analysis were performed. Forward logistic regression analysis was used to select potential contributing factors at a significance level of 5%. All statistical tests were two-tailed and were performed using SPSS V.20.0 software. The threshold for significance was a p-value <0.05.

## 3 Results

### 3.1 Clinical Data Characteristics of Patients

**Figure 1:**
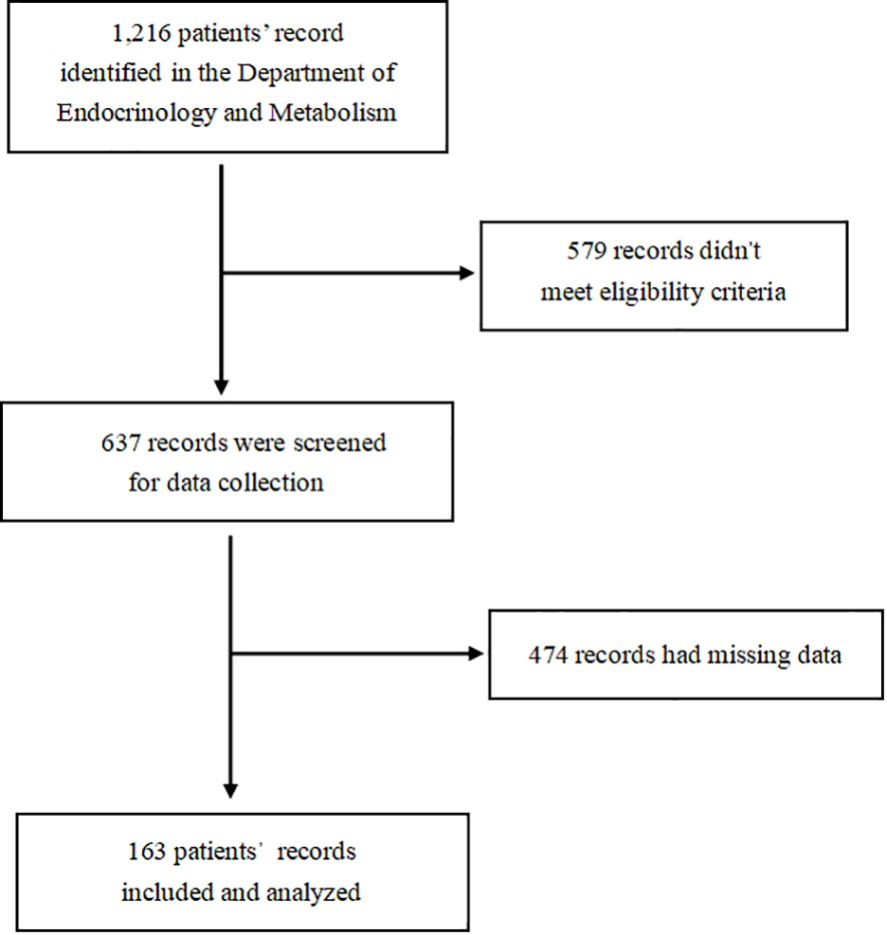
STROBE flow diagram for cross-sectional study.

This study is a retrospective cross-sectional study. Our method of screening patients was based on the time of admission in combination with our inclusion and exclusion criteria. In addition, the study of our collected indicators was carried out during the patients’ hospitalization. Some patients with incomplete data were excluded. Finally, a total of 163 patient files were included and analyzed (**Figure 1**).

The characteristics of the overall cohort are presented in **Table 1**. A total of 163 patients were included in the study, with a mean age of 57.46 ± 10.63 years and an almost equal gender distribution (50.31% men, 49.69% women). The average homocysteine (HCY) level, which is the primary focus of this study, was 14.53 ± 5.22 μmol/L. Physical examination results showed that the mean systolic blood pressure (sBP) and diastolic blood pressure (dBP) were 135.17 ± 16.23 mmHg and 82.09 ± 9.07 mmHg, respectively. The average body mass index (BMI) was 24.84 ± 3.47 kg/m², and the mean waist circumference was 90.46 ± 9.40 cm. Laboratory test results indicated that the average fasting glucose at 0 min was 9.23 ± 3.96 mmol/L, while the 120-min postprandial glucose was 17.59 ± 4.96 mmol/L. The average glycated hemoglobin (HbA1c) was 9.74 ± 2.42%, and the C-peptide levels at 0 min and 120 min were 2.05 ± 1.09 µg/L and 4.06 ± 2.38 µg/L, respectively. The mean serum creatinine (CR) was 66.74 ± 40.71 μmol/L, and the average uric acid (UA) level was 311.58 ± 93.29 μmol/L. The mean triglyceride (TG) level was 2.17 ± 1.58 mmol/L, while total cholesterol (TC) was 4.40 ± 1.15 mmol/L. The average high-density lipoprotein cholesterol (HDL-C) was 1.19 ± 0.33 mmol/L, and the low-density lipoprotein cholesterol (LDL-C) was 2.64 ± 0.77 mmol/L. Regarding urinary parameters, the average urinary creatinine (CR) level was 2429.33 ± 4354.32 mg/g, urinary microalbumin was 139.45 ± 562.51 mg/dL, and the urinary albumin-to-creatinine ratio (ACR) was 22.02 ± 97.45 mg/mmol. The mean high-sensitivity C-reactive protein (hs-CRP) level was 3.59 ± 6.95 mg/L. Carotid ultrasound results indicated that the mean right carotid intima-media thickness (IMT-R) was 0.96 ± 0.16 mm, while the left carotid intima-media thickness (IMT-L) was 0.97 ± 0.15 mm.

**Table 1:** Baseline characteristics in the overall cohort.

Parameters		Overall Cohort (n = 163)
Age(mean (SD)), y		57.46(10.63)
Gender(%)	Male	82(50.31)
	Female	81(49.69)
HCY(mean (SD)), µmol/l		14.53(5.22)
sBP(mean (SD)), mmHg		135.17(16.23)
dBP(mean (SD)), mmHg		82.09(9.07)
BMI(mean (SD)), kg/m2		24.84(3.47)
Waist circumference(mean (SD)), cm		90.64(9.40)
glucose 0min(mean (SD)),mmol/l		9.23(3.96)
glucose 120min(mean (SD)),mmol/l		17.59(4.96)
HbA1C (mean (SD)),%		9.74(2.42)
C peptide 0min(mean (SD)),µg/L		2.05(1.09)
C peptide 120min(mean (SD)),µg/L		4.06(2.38)
Cr(mean (SD)), μmol/L		66.74(40.71)
UA (mean (SD)), µmol/l		311.58(93.29)
TG (mean (SD)), mmol/l		2.17(1.58)
TC (mean (SD)), mmol/l		4.40(1.15)
HDL(mean (SD)), mmol/l		1.19(0.33)
LDL (mean (SD)), mmol/l		2.64(0.77)
Urine CR (mean (SD)), mg/g		2429.33 (4354.32)
Urinary microalbumin(mean (SD)),mg/dL		139.45(562.51)
UACR(mean (SD)), mg/mmol		22.02 (97.451)
hs CRP(mean (SD)),mg/l		3.59(6.95)
IMT-R(mean (SD)),mm		0.96(0.16)
IMT-L(mean (SD)),mm		0.97(0.15)

sBP, systolic Blood pressure; dBP, diastolic blood pressure; BMI, body mass index; CR, serum creatinine; hs CRP, high-sensitivity c-reactive protein; IMT, carotid intima-media thickness.

### 3.2 Clinical/biochemical characteristics across HCY

To investigate the association between homocysteine level and diabetic microvascular complications, including diabetic peripheral neuropathy and diabetic nephropathy, in type 2 diabetes patients, the data are presented in **Table 2**. In this study, 163 patients with type 2 diabetes were selected as observation group and were divided into three groups based on homocysteine level (10 umol/L and 15 umol/L as threshold). Analyze and compare the BMI, blood pressure, glucose tolerance, glycated hemoglobin, urinary microalbumin, urinary ACR, renal function, carotid artery intima-media thickness, and nerve conduction velocity of each group. The above results are listed in **Table 2**. The results showed that there were intergroup differences in gender, UA, CR, hs-CRP, and right tibial nerve motor nerve conduction velocity between different HCY groups (P < 0.05). No differences between groups were observed in other indicators.

**Table 2:** Baseline clinical/biochemical characteristics across HCY.

Clinical Data					
		HCY (<10) (n=22)	HCY (10-15) (n=82)	HCY (15-30) (n=59)	p-value
Age(mean (SD)), y		57.74(10.34)	56.95 (10.61)	59.2999.61)	0.481
Gender(%)	Male	13.64%	52.44%	61.02%	0.001
	Female	86.36%	47.56%	38.98%	
sBP(mean (SD)), mmHg		135.55(16.78)	134.66(15.71)	136.98(18.67)	0.909
dBP(mean (SD)), mmHg		82.59(3.18)	21.67(9.08)	84.11(9.42)	0.161
BMI(mean (SD)), kg/m2		25.03(3.41)	24.90(3.08)	25.31(3.92)	0.203
Waist circumference(mean (SD)), cm		91.293(9.19)	91.00(9.0805)	91.85(9.64)	0.067
UA (mean (SD)), µmol/l		318.74(93.97)	309.70(94.13)	329.04(93.84)	0.033
TG (mean (SD)), mmol/l		2.14(1.56)	2.02(1.51)	2.29(1.65)	0.304
TC(mean (SD)), mmol/l		4.22 (1.21)	4.40 (1.15)	4.44 (1.13)	0.664
HbA1C (mean (SD))		9.61(2.29)	9.84(2.48)	9.31(1.99)	0.082
HDL(mean (SD)), mmol/l		1.19(0.34)	1.22(0.39)	1.14(0.26)	0.711
LDL (mean (SD)), mmol/l		2.64(0.77)	2.62(0.79)	2.65(0.75)	0.805
CR (mean (SD)), μmol/L		66.36(32.71)	58.98(13.33)	78.16(49.68)	0.030
UACR(mean (SD)), mg/g		24.29 (101.492)	29.02(110.20)	18.15(89.73)	0.174
hsCRP(mean (SD)),mg/l		12.47 (14.93)	5.56 (7.75)	4.56 (5.68)	0.023
glucose 0min(mean (SD)),mmol/l		10.10 (6.25)	9.27 (3.27)	8.83 (3.84)	0.451
glucose 120min(mean (SD)),mmol/l		18.89 (7.06)	17.74 (4.45)	16.84 (4.73)	0.244
C peptide 0min(mean (SD)),µg/L		2.03 (1.14)	1.89 (1.03)	2.31 (1.11)	0.095
C peptide 120min(mean (SD)),µg/L		3.84 (2.05)	3.79(2.28)	4.78(2.42)	0.052
Urine creatinine(mean (SD)),mmol/L		3050.32(4134.85)	1994.63(3897.82)	2864.09(5016.67)	0.413
Urinary microalbumin(mean (SD)),mg/dL		22.31(27.60)	1.3023(339.94)	231.23(835.32	0.253
IMT-R(mean (SD)),mm		0.93(0.149)	0.97(0.152)	0.96(0.166)	0.564
IMT-L(mean (SD)),mm		0.95(0.150)	0.98(0.147)	0.97(0.166)	0.762
motor nerve of peroneal R(mean (SD)),m/s		44.16(0.69)	40.86(11.93)	43.15(5.00)	0.280
motor nerve of peroneal L(mean (SD)),m/s		43.67(5.74)	43.26(9.34)	41.92 (9.71)	0.667
motor nerve of median R(mean (SD)),m/s		50.00 (3.97)	50.90 (4.31)	52.00 (3.97)	0.284
motor nerve of median L(mean (SD)),m/s		50.73 (4.22)	51.13 (8.15)	52.46 (3.52)	0.572
motor nerve of ulnar R(mean (SD)),m/s		52.80 (4.76)	51.85 (11.09)	52.00 (4.68)	0.976
motor nerve of ulnar L(mean (SD)),m/s		53.50 (7.52)	51.46 (9.48)	53.16 (4.76)	0.597
motor nerve of tibial R(mean (SD)),m/s		41.07 (4.81)	45.38 (2.46)	43.78 (4.10)	0.031
motor nerve of tibial L(mean (SD)),m/s		40.71 (5.64)	42.68 (3.12)	42.79 (5.04)	0.321
sensory nerve of median R(mean (SD)),m/s		51.50 (6.07)	46.60 (11.41)	49.86 (6.05)	0.126
sensory nerve of median L(mean (SD)),m/s		51.42 (5.99)	46.18 (11.05)	48.97 (6.52)	0.125
sensory nerve of ulnar R(mean (SD)),m/s		47.67 (5.54)	43.83 (16.45)	49.33 (4.29)	0.183
sensory nerve of ulnar L(mean (SD)),m/s		49.33 (4.54)	46.33 (10.61)	50.35 (5.87)	0.084
sensory nerve of sural R(mean (SD)),m/s		40.45 (14.44)	42.48 (11.11)	41.61 (14.18)	0.799
sensory nerve of sural L(mean (SD)),m/s		40.16 (14.86)	41.17 (13.29)	41.46 (14.28)	0.942
sensory nerve of peroneal R(mean (SD)), m/s		47.00 (-)	40.54 (12.87)	45.33 (6.62)	0.486
sensory nerve of peroneal L(mean (SD)),m/s		45.00 (-)	40.31 (13.05)	43.75 (4.03)	0.656
Number of neuropathies(mean (SD))		3.76 (3.95)	4.65 (3.36)	3.72 (3.38)	0.247
Accumulated descent rate of neuropathy (mean (SD)),%		78.86 (96.36)	92.76 (86.60)	73.76 (78.29)	0.411
Average descent rate of neuropathy (mean (SD)),%		15.47 (8.93)	16.41 (7.42)	15.92 (7.46)	0.857

sBP, systolic Blood pressure; dBP, diastolic blood pressure; BMI, body mass index; CR, serum creatinine; hs CRP, high-sensitivity c-reactive protein; IMT, carotid intima-media thickness.

### 3.3 Simple Linear Regression analysis between Serum HCY Level and Other Indicators

**Figure 2:**
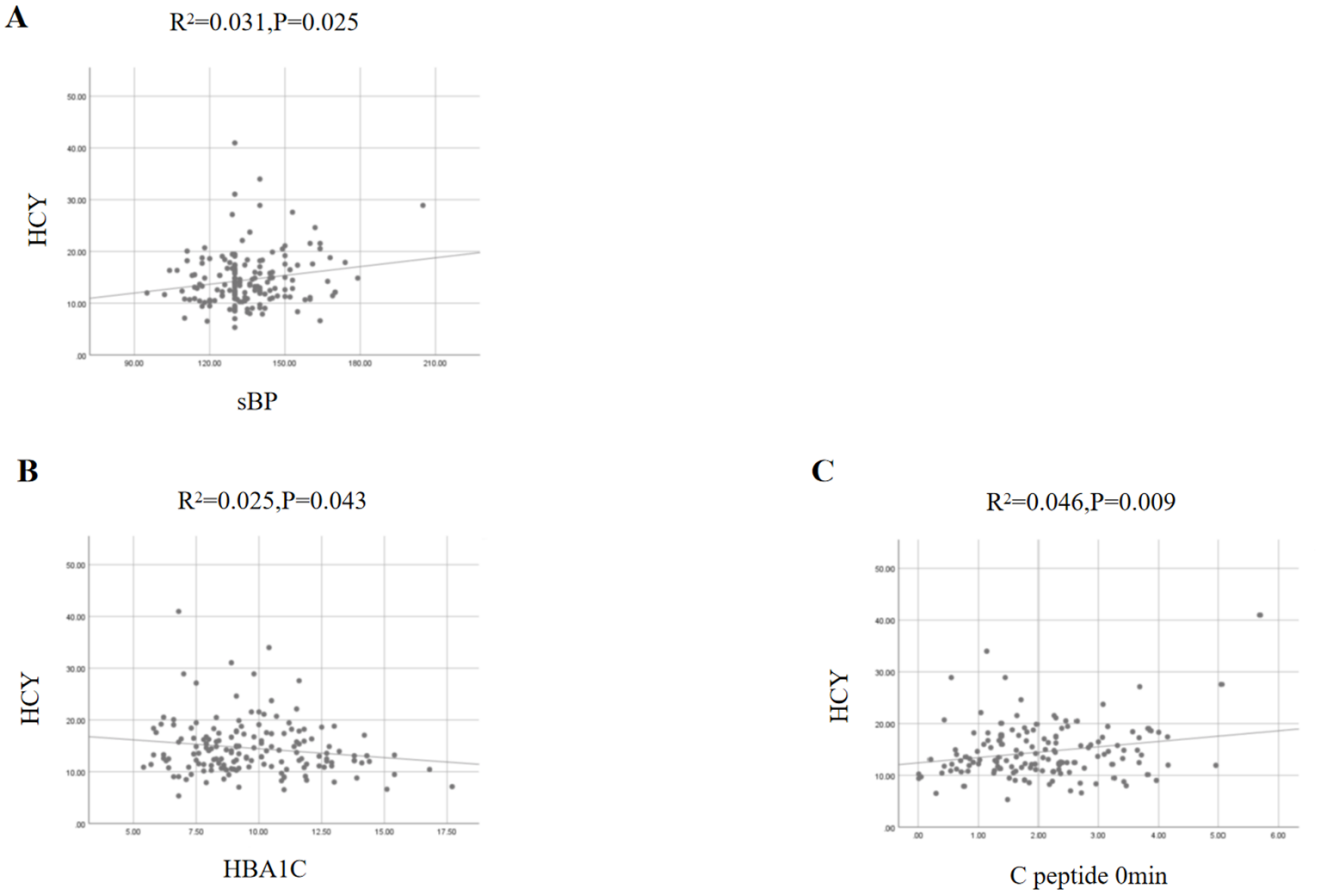
HCY and clinical examination indicators.

**Figure 3:**
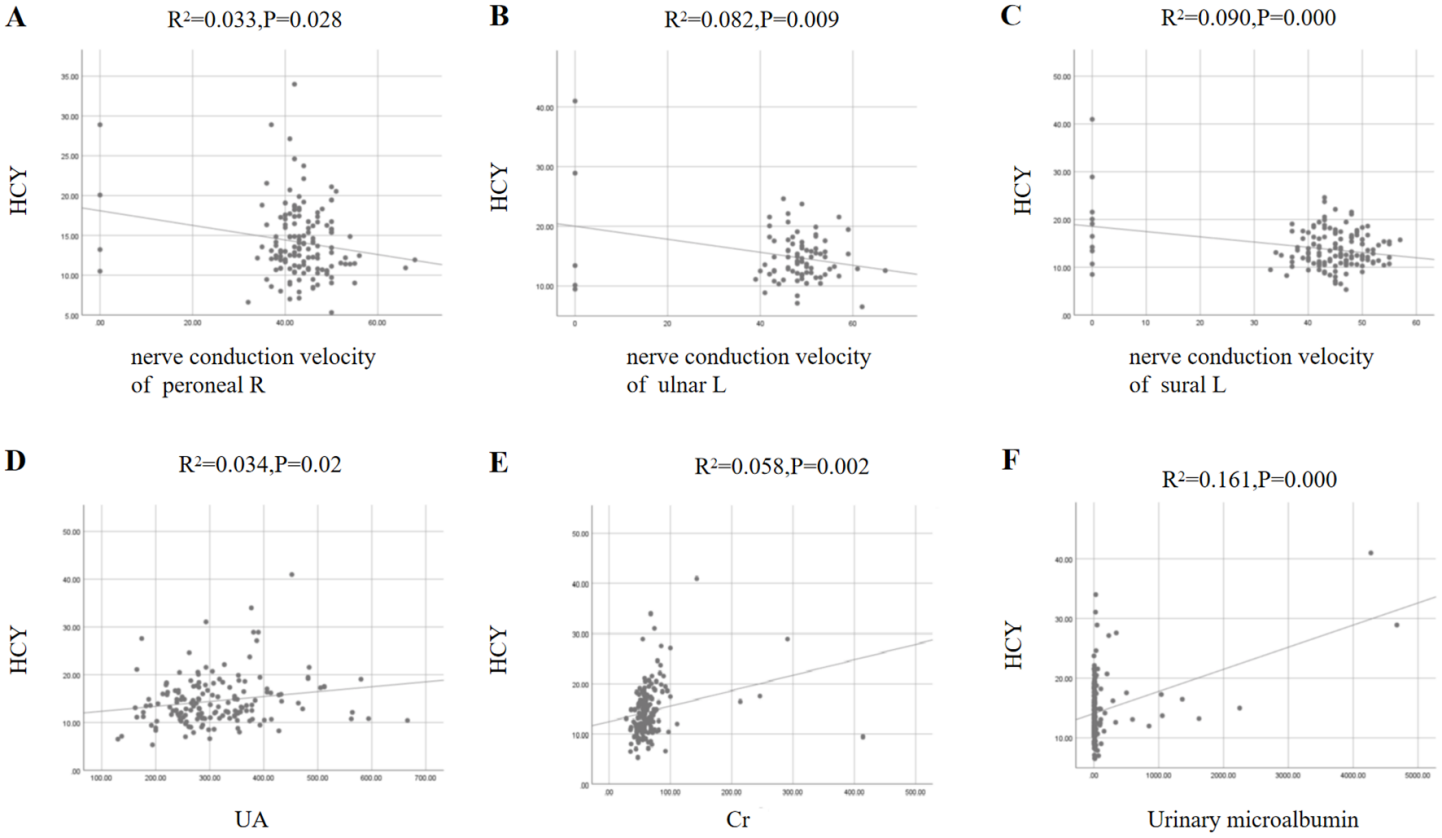
HCY and microvascular disease index of diabetes.

Simple linear regression analysis shows that HCY is correlated only with systolic blood pressure assessed in physical examination data (**Figure 2A**). In blood glucose-related detection indicators, HCY is associated with HBA1C (glycosylated hemoglobin) and C-peptide 0min (**Figures 2B, C**). Among the indicators of microvascular disease associated with diabetes, HCY correlates negatively with nerve conduction velocity of the common peroneal nerve, median nerve, and sural nerve and positively with creatinine, uric acid, and urinary microalbumin (**Figure 3**).

### 3.4 Stratified by Neuropathy and ACR, no difference in HCY was observed

**Figure 4:**
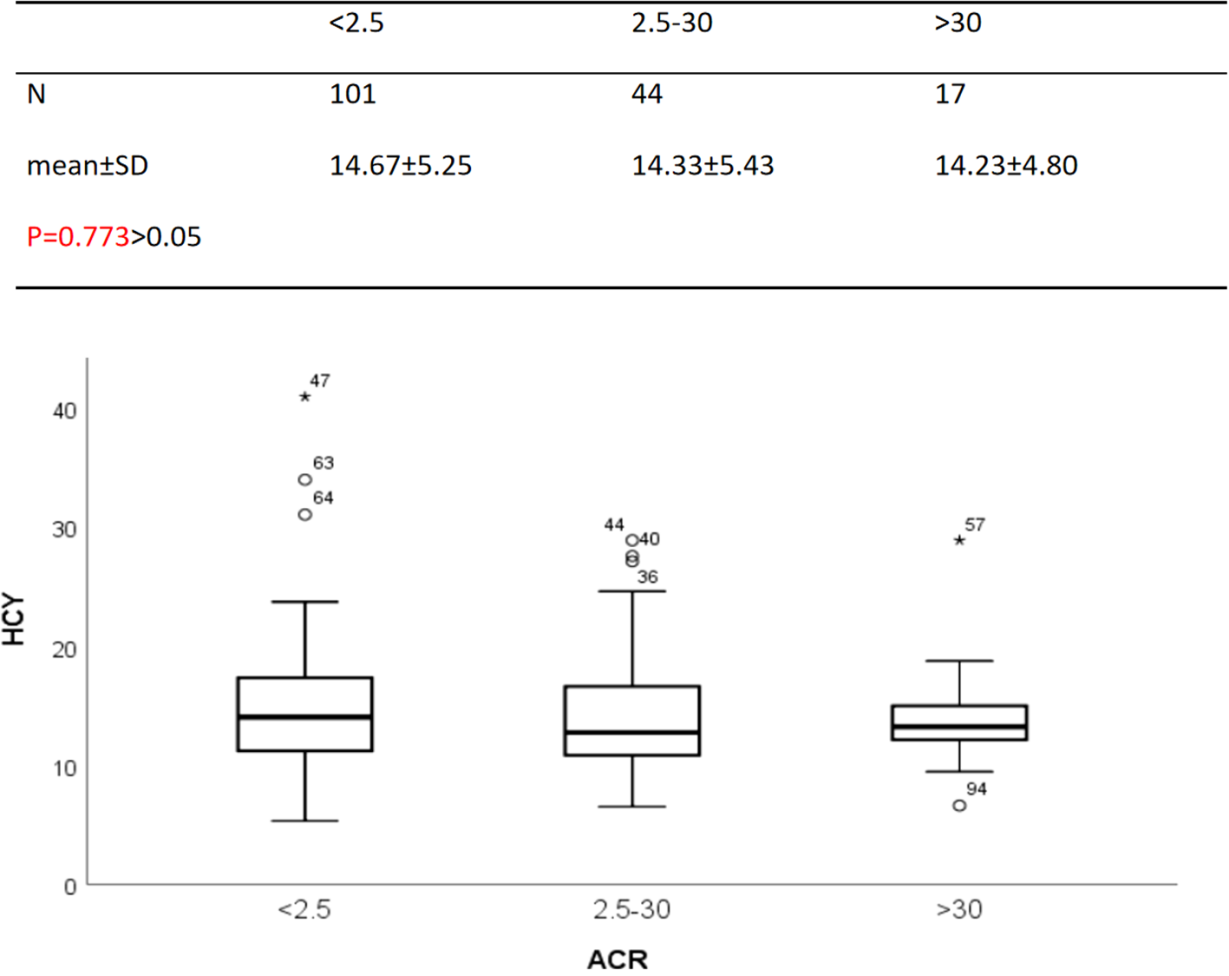
HCY values stratified by ACR.

**Figure 5:**
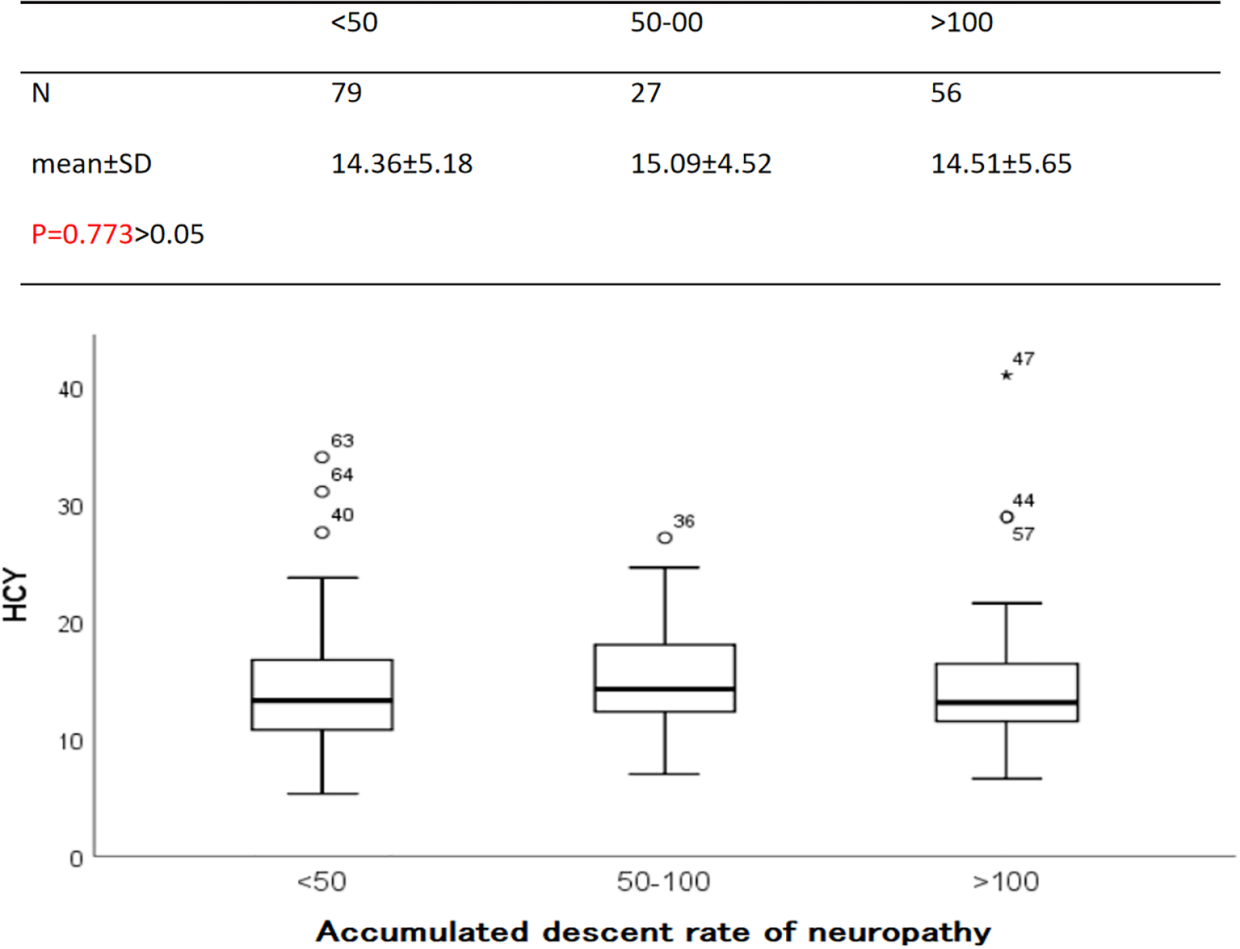
HCY values stratified by neural accumulated descent rate.

Since the correlation analysis shows that HCY is related to some indices of diabetic nephropathy and nerve conduction velocities, we would like to analyze whether there are differences between HCY and other indices according to the presence or absence of neuropathy (**Table 3**) or ACR (**Table 4**) are layered). Analysis shows that there is a difference in the average rate of nerve conduction decline observed in ACR stratification (P = 0.045). The median comparison of HCY stratified by ACR is shown in **Figure 4** (p = 0.773), the median comparison of HCY stratified by cumulative decline in nerve conduction velocity is shown in **Figure 5** (p = 0.457). In this study, no influence of neuropathy and ACR on HCY was found, and when stratified by the cumulative rate of decline in neuropathy, there was no difference in HCY. Stratified by urine ACR, there was also no difference in HCY.

**Table 3:** Comparison across neuropathy.

	P
HCY	0.894
Age(mean (SD)), y	0.773
Gender(%)	0.787
sBP(mean (SD)), mmHg	0.116
dBP(mean (SD)), mmHg	0.669
BMI(mean (SD)), kg/m2	0.488
Waist circumference(mean (SD)), cm	0.954
glucose 0min(mean (SD)),mmol/l	0.851
glucose 120min(mean (SD)),mmol/l	0.847
HBA1C(mean (SD)),%	0.692
C peptide 0min(mean (SD)),µg/L	0.110
C peptide 120min(mean (SD)),µg/L	0.109
Urine creatinine(mean (SD)),mmol/L	0.692
UA (mean (SD)), µmol/l	0.255
TG (mean (SD)), mmol/l	0.709
TC(mean (SD)), mmol/l	0.983
HDL(mean (SD)), mmol/l	0.722
LDL (mean (SD)), mmol/l	0.469
UACR(mean (SD)), mg/g	0.296
hsCRP(mean (SD)),mg/l	0.820
IMT-R (mean (SD)),mm	0.541
IMT-L (mean (SD)),mm	0.354
motor nerve of peroneal R(mean (SD)),m/s	0.000
motor nerve of peroneal L(mean (SD)),m/s	0.000
motor nerve of median R(mean (SD)),m/s	0.000
motor nerve of median L(mean (SD)),m/s	0.381
motor nerve of ulnar R(mean (SD)),m/s	0.499
motor nerve of ulnar L(mean (SD)),m/s	0.021
motor nerve of tibial R(mean (SD)),m/s	0.120
motor nerve of tibial L(mean (SD)),m/s	0.007
sensory nerve of median R(mean (SD)),m/s	0.110
sensory nerve of median L(mean (SD)),m/s	0.142
sensory nerve of ulnar R(mean (SD)),m/s	0.103
sensory nerve of ulnar L(mean (SD)),m/s	0.176
sensory nerve of sural R(mean (SD)),m/s	0.030
sensory nerve of sural L(mean (SD)),m/s	0.039
sensory nerve of peroneal R(mean (SD)), m/s	0.572
sensory nerve of peroneal L(mean (SD)),m/s	0.461
Number of neuropathies(mean (SD))	0.000
Average descent rate of neuropathy (mean (SD)),%	0.000

sBP, systolic Blood pressure; dBP, diastolic blood pressure; BMI, body mass index; CR, serum creatinine; hs CRP, high-sensitivity c-reactive protein; IMT, carotid intima-media thickness.

**Table 4:** Comparison across UACR.

	P
HCY	0.676
Age(mean (SD)), y	0.457
Gender(%)	0.418
sBP(mean (SD)), mmHg	0.667
dBP(mean (SD)), mmHg	0.574
BMI(mean (SD)), kg/m2	0.411
Waist circumference(mean (SD)), cm	0.286
glucose 0min(mean (SD)),mmol/l	0.594
glucose 120min(mean (SD)),mmol/l	0.248
HBA1C(mean (SD)),%	0.412
C peptide 0min(mean (SD)),µg/L	0.378
C peptide 120min(mean (SD)),µg/L	0.338
Urine creatinine(mean (SD)),mmol/L	0.262
UA (mean (SD)), µmol/l	0.186
TG (mean (SD)), mmol/l	0.634
TC(mean (SD)), mmol/l	0.060
HDL(mean (SD)), mmol/l	0.601
LDL (mean (SD)), mmol/l	0.052
hsCRP(mean (SD)),mg/l	0.534
IMT-R (mean (SD)),mm	0.329
IMT-L (mean (SD)),mm	0.867
motor nerve of peroneal R(mean (SD)),m/s	0.684
motor nerve of peroneal L(mean (SD)),m/s	0.790
motor nerve of median R(mean (SD)),m/s	0.062
motor nerve of median L(mean (SD)),m/s	0.502
motor nerve of ulnar R(mean (SD)),m/s	0.144
motor nerve of ulnar L(mean (SD)),m/s	0.525
sensory nerve of ulnar R(mean (SD)),m/s	0.927
sensory nerve of ulnar L(mean (SD)),m/s	0.502
sensory nerve of peroneal R(mean (SD)), m/s	0.144
sensory nerve of peroneal L(mean (SD)),m/s	0.528
Number of neuropathies(mean (SD))	0.260
Accumulated descent rate of neuropathy (mean (SD)),%	0.173
Average descent rate of neuropathy (mean (SD)),%	0.045

sBP, systolic Blood pressure; dBP, diastolic blood pressure; BMI, body mass index; CR, serum creatinine; hs CRP, high-sensitivity c-reactive protein; IMT, carotid intima-media thickness.

## 4 Discussion

This study further supports the current argument that HCY levels are associated with type 2 diabetic nephropathy and diabetic peripheral neuropathy. A total of 163 type 2 diabetics were included in this study. Comparison of HCY groups across groups indicated that there were differences between genders and the proportion of men was higher in the group with HCY > 15 umol/L (P = 0.001), consistent with the conclusion that the HCY levels were relatively higher in men (13). A comparative analysis between groups also showed that the higher HCY group had higher levels of uric acid (P = 0.033) and creatinine (P = 0.030). Linear regression analysis showed a positive correlation with urinary uric acid (R2 = 0.034, P = 0.02), creatinine (R2 = 0.058, P = 0.002) and urinary microalbumin (R2 = 0.161, P = 0.000). HCY negatively correlates with nerve conduction velocity: right peroneus (R2 = 0.033, P = 0.028), left ulna (R2 = 0.082, P = 0.009), left sural (R2 = 0.090, P = 0.000). Stratified by urine ACR values, it can be seen that there were differences in the average rate of nerve degradation (P = 0.045). The above results demonstrate that HCY is associated with microvascular diseases of type 2 diabetes – diabetic peripheral neuropathy and diabetic nephropathy. Furthermore, although we found differences in tibial nerve conduction velocity between the HCY groups, we did not observe a decrease in tibial nerve conduction velocity in the high HCY level group. This may be because we did not collect enough data on tibial nerve conduction velocity.

China is the country with the highest rate of diabetes (14). The prevalence of various complications in T2DM patients in China is high: the survey of 14,289 T2DM patients hospitalized in endocrine or diabetes hospitals (mean age 60.5 years, mean course 9.0 years) shows that microvascular and Macrovascular complications are common in diabetic patients (15). Microangiopathy in diabetes is a common chronic complication of diabetes, mainly manifested by diabetes retinopathy, diabetes nephropathy and diabetes neuropathy. These microvascular complications significantly affect patients’ quality of life. Microvascular complications are an important cause of mortality and disability in patients (16–18). Among the serious complications of diabetes, diabetic neuropathy has the highest prevalence and serious damage. Diabetic peripheral neuropathy (DPN) is a loss of sensory function that begins at the distal end of the lower limbs and is also characterized by pain and a severe incidence rate. 50–75% of non-traumatic amputations are caused by DPN, and 68.1% of diabetic patients die within 5 years of amputation (19, 20). More than a third of diabetes patients in China suffer from complicated chronic kidney disease (21). Once diabetic patients develop complicated chronic kidney disease, the risk of adverse events and all-cause death doubles (22). Proteinuria is a sensitive indicator for detecting early renal lesions and a predictive factor for the rapid progression of chronic kidney disease (23, 24). In our study, differences in neuropathy were observed by stratification of urinary ACR lesions (**Table 4**, P=0.045). This suggests that the two types of lesions are microvascular lesions with the same pathogenesis and similar severity. Given the side effects of diabetes complications, an early prevention and intervention method is urgently needed.

Homocysteine (Hcy) is an important metabolite in methionine metabolism. When homocysteine’s metabolic pathway is abnormal, it accumulates in the body, ultimately leading to hyperhomocysteinemia. In recent years, many studies have found that hyperhomocysteinemia is related to the occurrence and development of various diseases such as glaucoma (25), Parkinson’s disease (26), arteriosclerosis (27), cancer (28) and metabolic syndrome (29), chronic obstructive pulmonary disease (30). In order to change the treatment status of diabetes, peripheral neuropathy and diabetes, many scientists also focus on HCY. The research by Ning Ma et al. suggested that plasma tHcy concentrations are relatively elevated in elderly patients with DKD, especially those aged ≥75 years, and that tHcy could serve as a biomarker for the development of DKD in elderly patients, but this was not ideal for prediction of T2DM (31). The research by Ma L et al. suggested that the increase in circulating homocysteine concentration has a causal relationship with the increased risk of DKD in Chinese patients with diabetes (32). The research by Ding S et al. suggested that the levels of HCY and NRG4 are closely related to the severity of DKD in T2DM patients with early DKD. The combined HCY/NRG4 detection can detect the occurrence of DKD in diabetics at an early stage (11). In a retrospective study, patients were divided into two groups based on blood Hcy levels, namely the HHcy group and the NHHcy group. The incidence rates of DPN in both groups were 98.5% and 36.2%, respectively. Correlation analysis was used to examine the correlation between Hcy level and the incidence rate of DPN. The results showed a significant correlation between Hcy and DPN (33). There are also studies suggesting that glycated hemoglobin (HbA1c) and homocysteine (HCY) are closely related to DPN (34).

Our results also support the view that elevated homocysteine concentration is associated with diabetic peripheral neuropathy and diabetic nephropathy. However, glucose control can effectively prevent the progression of diabetic neuropathy in type 1 diabetes patients, but the effect is weak in type 2 diabetes patients (35). This also partially explains that in our study there was no difference in blood glucose (including fasting blood glucose, postprandial hourly blood glucose, glycated hemoglobin) when stratified by neuropathy (**Table 3**). In addition, our research also has some shortcomings, such as: B. fewer recorded cases and no statistics about the course of the patients. Furthermore, we did not further observe whether reducing homocysteine can improve nerve conduction velocity and outcome of diabetic nephropathy.

## 5 Conclusions

DPN and DKD are common microvascular complications of diabetes. This study was conducted to discover the functions of HCY in DPN and DKD. Our results confirmed the positive correlations of HCY and DPN and DKD. Further studies with larger cohorts of participants should focus on the possible mechanisms and therapeutic effects of HCY on DPN and DKD.

## Data Availability

The original contributions presented in the study are included in the article/Supplementary Material. Further inquiries can be directed to the corresponding authors.
